# Modeling the population dynamics of lemon sharks

**DOI:** 10.1186/1745-6150-9-23

**Published:** 2014-11-18

**Authors:** Easton R White, John D Nagy, Samuel H Gruber

**Affiliations:** School of Life Sciences, Arizona State University, P.O. Box 874501, 85287 Tempe, USA; Department of Life Sciences, Scottsdale Community College, Chaparral Road, Scottsdale, 85256 USA; School of Mathematical and Statistical Sciences, Arizona State University, P.O. Box 871804, 85287 Tempe, USA; Bimini Biological Field Station, Bimini, Bahamas; Center for Population Biology, University of California - Davis, Davis, CA USA

**Keywords:** Demography, Density-dependence, Elasmobranch, Inverse modeling, Population dynamics, Stage-based, Stochasticity

## Abstract

**Background:**

Long-lived marine megavertebrates (e.g. sharks, turtles, mammals, and seabirds) are inherently vulnerable to anthropogenic mortality. Although some mathematical models have been applied successfully to manage these animals, more detailed treatments are often needed to assess potential drivers of population dynamics. In particular, factors such as age-structure, density-dependent feedbacks on reproduction, and demographic stochasticity are important for understanding population trends, but are often difficult to assess. Lemon sharks (*Negaprion brevirostris*) have a pelagic adult phase that makes them logistically difficult to study. However, juveniles use coastal nursery areas where their densities can be high.

**Results:**

We use a stage-structured, Markov-chain stochastic model to describe lemon shark population dynamics from a 17-year longitudinal dataset at a coastal nursery area at Bimini, Bahamas. We found that the interaction between delayed breeding, density-dependence, and demographic stochasticity accounts for 33 to 49% of the variance in population size.

**Conclusions:**

Demographic stochasticity contributed all random effects in this model, suggesting that the existence of unmodeled environmental factors may be driving the majority of interannual population fluctuations. In addition, we are able to use our model to estimate the natural mortality rate of older age classes of lemon sharks that are difficult to study. Further, we use our model to examine what effect the length of a time series plays on deciphering ecological patterns. We find that—even with a relatively long time series—our sampling still misses important rare events. Our approach can be used more broadly to infer population dynamics of other large vertebrates in which age structure and demographic stochasticity are important.

**Reviewers:**

This article was reviewed by Yang Kuang, Christine Jacob, and Ollivier Hyrien.

## Background

Many large marine megavertebrates (e.g. sharks, turtles, mammals, seabirds) are particularly vulnerable to anthropogenic mortality due to their complex life history characteristics, including long lifespans, delayed maturity, low fecundity, and extended migrations [1–4]. These animals often act as ecological keystones, and their removal can lead to considerable ecosystem changes such as cascading ecological effects on lower trophic levels [5–9]. For example, as predators, sharks not only regulate their own prey populations but also those of species deeper in the food web [5, 7–9] (see also [10] for a recent review). Given their importance to ecosystem stability and the multiple anthropogenic threats they face [11, 12], it is imperative that we develop a better understanding of shark population dynamics, particularly to identify primary drivers of annual population variation.

Physiologically structured population models [13, 14] that incorporate delayed breeding [15, 16], density-dependent mechanisms [13, 17], demographic stochasticity [18–22], or some combination of these processes, have been applied to many ecological systems to answer questions related to population dynamics, conservation, and management. In examining shark populations, physiologically structured discrete demographic models have been used to study overfishing and population viability, calculate specific demographic parameters, and predict population dynamics [23–30], also see review [31]. Although these demographic models are useful, they typically have three key shortcomings [31]: (1) they typically include assumptions that are biologically unrealistic, including density independent, deterministic mechanisms, which makes application of model outputs to real data difficult to accept; (2) given that these models are deterministic, they cannot capture demographic stochastic events, which are likely to be important drivers of interannual population fluctuations; and (3) parameterization and validation of such models from data are often logistically difficult and require long-term field operations [2, 32].

**Figure 1:**
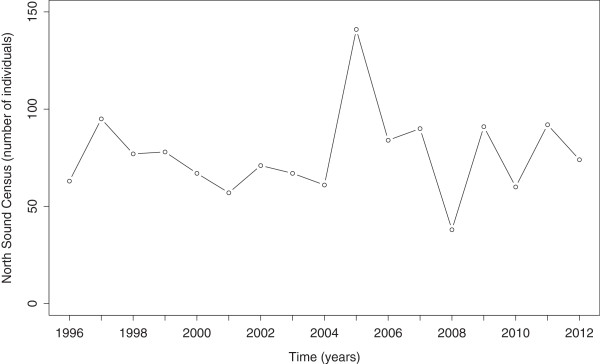
**Juvenile population data from the past 17 censuses in the North Sound of Bimini.**

The third challenge has been met by a longitudinal field study of lemon sharks (*Negaprion brevirostris*) at Bimini, Bahamas. Data from this study includes an annual population census of juvenile lemon sharks (ages 0-2 years) from 1996 to 2012. The number of juveniles in our study population (see Methods) typically fluctuates between 50 and 100 sharks, although the complete range is estimated to be between about 35 and 150 (Figure 1), which illustrates the significance of annual fluctuations in the juvenile age class of this lemon shark population. Fecundity and early juvenile mortality rates have been estimated precisely using mark-recapture and genetic methods [33–35].

The causes of annual variation in population size remain unclear for many species, and we are unaware of any previous studies that have assessed these causes in detail for lemon sharks. Furthermore, little is known regarding mortality rates of both the larger juveniles that have left the nursery site (ages 3-11) and mature adults (ages 12+) [36, 37].

Here we present a mathematical model detailing annual dynamics of the lemon shark population in Bimini, Bahamas. The model is physiologically structured, with age class as the (discrete) structuring variable. Although the model tracks all age classes, the population of juveniles is the only observable connection to the real population. The model is similar to that of Hoenig and Gruber (1990), who studied a deterministic Leslie matrix model. Here, we introduce demographic stochasticity by making births and deaths stochastic, although we fix environmental parameters. We use inverse pattern-oriented techniques that leverage fecundity and juvenile mortality rate estimates obtained from the field study described above to obtain bounds on unobserved parameters, particularly adult mortality, with a minimum of assumptions extraneous to the model.

## Results

The Bimini nursery data suggest that, on average, about 77 juvenile sharks inhabit the lagoon at census time, with interannual variance, *s*^2^≈498 (Figure 1). Our age-structured stochastic model depends on 3 parameters: mean litter size (denoted *λ*), adult mortality (*μ*) and a parameter controlling density dependent feedback of number of juveniles on juvenile mortality (denoted *k*, which measures the effect of competition among juveniles in the nursery; larger *k* means less effect of competition; see Methods for details and Table 1 for a summary of notation). Setting these parameters to the following values—(litter size) *λ*=6.1 sharks per female, (adult mortality probability) *μ*=0.15, (density-dependent feedback) *k*=100 sharks—generates a mean juvenile population size in model simulations that matches that observed in the field; however, at these parameter settings the model fails to generate the proper variance. Using the Monte Carlo technique described in the Methods section, we generated an estimate of the model’s sampling distribution of the variance for samples of size 17 years. The mean of this distribution of variances, denoted , is 176 (*n*=100 trials), which represents only 35 percent of *s*^2^ in the actual data set.

**Table 1:** **Notation and interpretations of model parameters, their default values, ranges and sources for the lemon shark (** ***Negaprion brevirostris*** **)**

Parameter	Meaning	Default	Range	Source
*λ*	Pups born per female	6.1	1-18	[34, 35]
*h*	Juvenile mortality Hill parameter	1	NA	This paper
*k*	Juvenile mortality shape parameter	100	0-200	This paper
*t*_*max*_	Maximum age for adult	25	20-35	[23, 26]
*x*_*m*_	Age at maturity	12	NA	[23, 26]
*μ*	Mortality rate for all animals above age one	0.15	0.05-0.30	This paper

**Figure 2:**
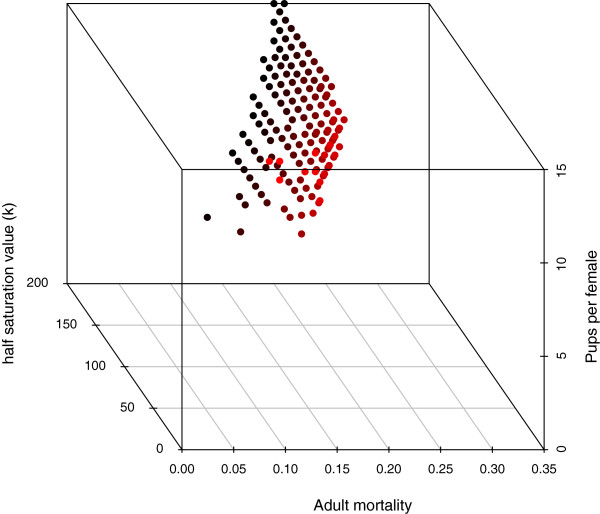
**Region of parameter space in which simulations exhibited a “good” fit to the data of the lemon shark population based on criteria described in the main text.** Each filled circle represents one of the 9000 parameter combinations that met the criteria of a good representation. The change in color represents degree of half saturation value, with red indicating smaller values of *k*.

**Figure 3:**
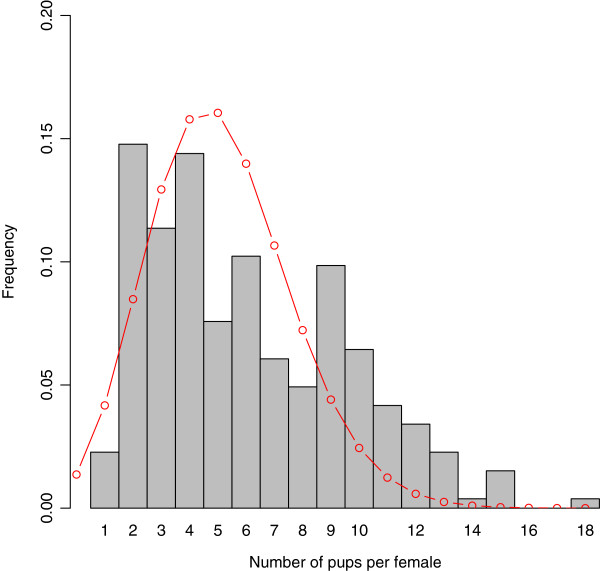
**Distribution of litter size per female lemon shark in North Bimini.** Grey bars: data from [34, 35], from 1996 to 2010 (*n*= 264). Red curve: discrete Poisson distribution, , with *λ* equal to the mean of the litter size distribution depicted by the grey bars.

**Figure 4:**
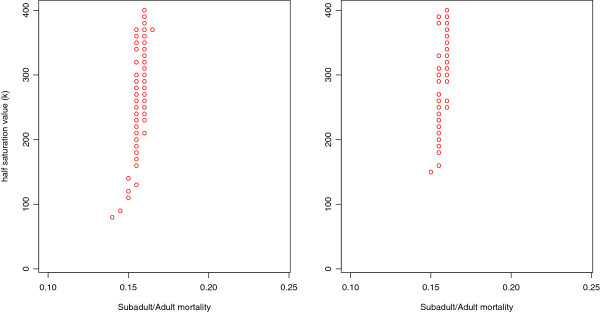
**Circles represent region of the parameter space in which simulations were a “good” fit to the data (see**Methods**).** Left: Half-saturation value (*k*, density-dependence parameter for the first-age class mortality rate) versus the mortality rate for subadults and adults for series of combinations utilizing the actual distribution of litter sizes for fecundity rate. Right: Same as left but uses a Poisson distribution for fecundity rates. Both pictures represent cases when *λ* was set at 6.1 for the Poisson distribution which is equivalent to the average of the actual distribution of litter sizes.

To explore the sensitivity of mean and variance to variations in parameters, we systematically tested 9000 possible combinations of the three model parameters (*λ*, *μ*, *k*) throughout a generous region of parameter space that contained biologically plausible values for all parameters (see Methods). For each of the 9000 points in parameter space, we compared field (census) estimates of annual mean and variance of population size to annual mean and variance of simulated population size. As above (and described fully in Methods), each of the 9000 points in parameter space was associated with a Monte Carlo estimate of the sampling distributions for mean and variance for samples of size 17. For each parameter combination we performed a 2-tailed Monte Carlo test of the observed mean and variance against their estimated sampling distributions generated by the model. For ease of exposition, we call the fit “good” if neither observed mean nor variance fell in the rejection region of the sampling distribution and the other criteria explained in the Methods were met; otherwise, the fit was deemed “bad”. At most, but not all, of the tested points, the fit was bad. Therefore, the volume of the possible parameter space that admits dynamics having any chance of representing the actual Bimini population is greatly constrained (Figure 2), even when litter size is allowed to vary from 1-15. As described in the Methods, we considered two possible distributions of litter sizes: one Poisson and the other derived from field data. In Figure 2 we show the results assuming litter sizes were Poisson-distributed according to equation (3). However, using the observed distribution of births per female (Figure 3) instead of the Poisson assumption further tightens our constraints on the mortality parameters *k* and *μ* (Figure 4).

For combinations that fit the data based on our criteria, we found that, while demographic stochasticity alone can correctly predict the mean population size in a sizable region of parameter space, at best it can account for only 33% to 49% of the observed variance—nowhere in parameter space was the observed variance in the inner quartile range of the sampling distribution of the variance. These results suggest that either the model is valid and our sample happened to have an extremely unlikely variance, or the model is not valid and something in addition to demographic stochasticity is generating population fluctuations.

This sensitivity analysis also allows us to use inverse pattern-oriented techniques (see Methods) to obtain bounds on the parameters that may be used to generate prior probabilities for Bayesian analysis of a more complete model. The pattern-oriented analysis places tight constraints on adult mortality (*μ*=0.14−0.17), which generally agrees with the indirect methods for estimating mortality in this population [38–40] (Table 2), which place mortality between 0.086-0.179. Interestingly, the half-saturation value (*k*) is much less constrained; good fits can be obtained for any *k*>100 (Figure 4).

**Table 2:** **Indirect methods used to calculate mortality rates**

Method	Relationship	Value
Hoenig (1983) (fish)	*l* *n*(*Z*)=1.46−1.01 *l* *n*(*t*_*max*_)	0.167
Hoenig (1983) (cetacean)	*l* *n*(*Z*)=0.941−0.873 *l* *n*(*t*_*max*_)	0.154
Hoenig (1983) (combined)	*l* *n*(*Z*)=1.44−0.982 *l* *n*(*t*_*max*_)	0.179
Pauly (1980)	*l* *o* *g*(*M*)=−0.0066−0.279 *l* *o* *g*(*L*_*∞*_)	
	+0.6543 *l* *o* *g*(*K*)+0.4634 *l* *o* *g*(*T*)	0.140
Jensen (1996) (age)	*M*=1.65/*x*_*m*_	0.138
Jensen (1996) (growth)	*M*=1.5 *K*	0.086
Jensen (1996) (Pauly)	*M*=1.6 *K*	0.091

Here *M* and *Z* represent natural and total mortality, respectively. Similar analysis as [41] and [42]. Note: Life history parameters are based on [36]. *K*, body growth parameter (0.057); *L*_***∞***_, maximum theoretical length (317.65 cm); *x*_*m*_, age at maturity (12 years); *t*_*max*_, maximum age (25); T, mean temperature (27.1°C, [43]).

It is important to note that even for parameter combinations that qualified as good fits, all greatly underestimated annual variance observed in the actual data.

In general, our model dynamics were robust with respect to the two assumed distributions of per-female fecundity—either Poisson or empirical. Specifically, both produced very similar regions of parameter combinations that matched Bimini (Figure 4), with the nuanced exceptions noted above. In addition, however, the actual distribution of litter sizes consistently generated a higher variance in annual population size than did the Poisson distribution. Therefore, although the Poisson distribution is a reasonable choice to use when the actual distribution is not available, one needs to be aware that it tends to underestimate variance.

**Figure 5:**
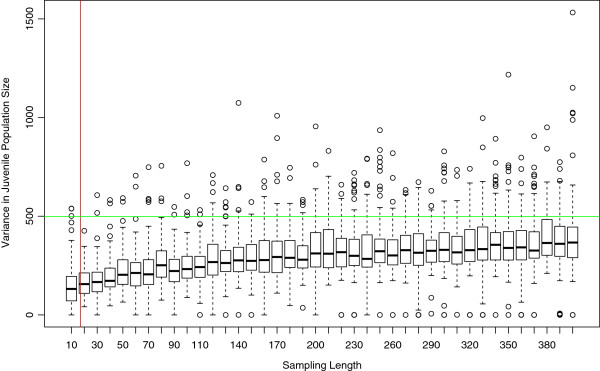
**Boxplots of simulation variance as a function of the length of study period (sample size).** The sample at Bimini is a total of 17 years (indicated by the vertical red line). The green line represents the variance in the actual population size (*s* ^2^=498).

As a further test of how well our model represents the variance in juvenile population size, we examined the effect of study length (or sample size) on characterization of the observed variance. We compared time intervals of various lengths by generating sampling distributions of the variance for samples of various sizes, from 10 to 400 (Figure 5). The median of the estimated sampling distribution of the variance is a generally increasing function of sample length, as expected since longer samples have a higher probability of capturing rare demographic events (e.g., exceptionally large number of pups across all females or high mortality by chance). Notice that, even for “studies” of hundreds of years, the observed variance is always well into the upper tail of the estimated sampling distributions. So, even though the median variance is higher in 400 compared to 17 year samples (vertical red line in Figure 5), the observed variance of 498 sharks ^2^ (horizontal green line in Figure 5) would still be remarkably large for much larger samples. This further supports our conclusion that the model fails to match the observed variance, and that the deviation between expectation from the model and observation in the field is caused by something other than random chance.

## Discussion

Lemon sharks have complex life histories—they delay breeding for over a decade, mature in an environment (nursery lagoons at the Bimini site) vastly different from their adult habitat (open ocean) and when mature breed every other year. Also, nursery populations are typically not large enough (order 10^2^ at most) to buffer demographic stochasticity; indeed, demographic stochasticity can dominate dynamics in patchy systems with sizes orders of magnitude larger than this one [44–46].

Therefore, generalized, deterministic population models can hope to elucidate only the broadest outlines of lemon shark population dynamics and should be interpreted only in the “ensemble average” sense [44, 47]. That is, deterministic models at best provide an expectation or mean behavior for an infinite number of Bimini’s lemon shark populations. Although this abstract notion of an ensemble mean is sensible and provides some insight about expected behavior of the population, under a suitable definition of “expected”, that insight is limited because such models provide no measure of the fluctuations about this ensemble average one can expect to see in any real instance [19].

We addressed this shortcoming by developing a model of the lemon shark population at Bimini incorporating both demographic stochasticity and age structure. Despite the added realism, the model remains relatively simple. Parameters requiring estimates include the probability distribution for the number of pups born to breeding females in a give year, or just the mean number of pups per female if one assumes a Poisson distribution, two parameters characterizing density-dependent mortality in the first age class, and the probability(-ies) of mortality for all individuals in all other age classes, which here we assume to be invariant across individuals. We obtain the reproductive parameters directly from a field study of the Bimini lemon shark nursery (Figure 3), and we use an exhaustive parameter search to obtain bounds on the other parameters.

### Fecundity assumptions

As far as we know, this is the first elasmobranch study to use actual litter sizes derived from genetic data to parameterize a mathematical or computational model’s birth function. Typically such studies rely on an assumed distribution, of which Poisson is usually thought to be a good first estimate. Since we have a data-driven, realistic estimate of the distribution of litter sizes, we can explore the consequences of making the Poisson assumption, and we find that, in this instance, the Poisson assumption appears reasonable. If researchers only have mean number of pups per female, which is often the case without genetic maternity data, the Poisson distribution approximates the actual distribution remarkably well (Figure 3). Also, the regions of viability within the parameter space for both actual and Poisson distributions are similar (Figure 4). However, the simulations we ran using the Poisson assumption consistently exhibited lower interannual variance than did simulations using the actual reproductive data. Therefore, although the Poisson assumption generates estimates of adult mortality that are essentially identical to those produced using the data, it should be used with care when modeling to assess population viability and fluctuations.

### Mortality assumptions

In the present study we model density-dependent mortality in the first age class as a nonstationary Bernoulli process; that is, the probability of mortality is a generally increasing function of the number of sharks in this age class. This assumption is justified based on field data [28, 33]. We represented this density-dependent mortality using a Hill function (equivalently a Michaelis-Menten form) from phenomenological considerations only—we hypothesize a monotonic but saturating increase in mortality with density, which the Hill function exhibits, with the added benefits of relative simplicity and plasticity. Nevertheless, the parameters of this function have biological meaning. In particular, the shape parameter, *k*, measures how sensitive sharks are to competition from same-age conspecifics. As such, although it quantifies an important biological response, in general it will be very difficult to estimate accurately in the field. Importantly, this model demonstrates that practical estimates need not be very precise. The model matches data for a wide range of this parameter’s values; in fact, for *k*>100 or so, the model fit is largely unaffected (Figure 4). Therefore, this model is robust for parameterizations of *k*.

The bounds we found for subadult and adult mortality rates are remarkably narrow (Figures 2 and 4). In fact, if demographic stochasticity is the only cause of fluctuations, this model predicts that the probability of mortality for any individual in any age class, after the first, lies between 0.14 and 0.17. Indeed, above 0.17, populations invariably die out very rapidly. These mortality estimates compare favorably to estimates obtained using the techniques in Table 2. If subadult and adult mortality were to increase by only a few percentage points, the model predicts rapid extinction. Therefore, we suggest that any added fishing pressure (there is currently no fishing pressures for lemon sharks at Bimini) to this population would threaten its sustainability. This result agrees with others in suggesting that long-lived species with low fecundity, like lemon sharks, would not be able to handle added fishing mortality in adult age classes [48].

One difficulty this model faces is a lack of information about mortality in subadults and adults. As a first approximation, we assume a fixed probability of mortality in all age classes after the first. However, we recognize the tentative nature of this assumption. In particular, it is challenging to relate age to mortality rate of untagged adult sharks. Peterson and Wroblewski [49] suggested that one method to overcome this problem is to construct a function that maps shark mortality rate to mass. In this case age can then be related to mass with conversion methods such as those used in [27]. This method needs modifications in situations where mortality is density-dependent, and it relies on assumptions of von Bertalanffy growth and reliable estimate of mass, length, and age from catch data. However, where applicable, this technique may only be needed for the first few age classes, or at least until the age at which individual sharks are large enough avoid being preyed upon by natural predators [24]. However, in this study such a technique is unavailable because size data of adult sharks is limited to length only; length-to-weight standards exist only for juvenile age classes (Gruber, unpublished data).

### Effect of sampling length

As one might expect, we found that a longer time series (i.e., sampling length or more years sampled) from simulated populations represent the actual population better than smaller samples do. The quality of the representation, as measured by a comparison of observed variance in population size to the estimated sampling distribution of variance from simulations, appears to approach some parametric value asymptotically (Figure 5). This asymptotic approach requires samples measured in units of centuries (Figure 5). Apparently, random walks to exceptionally large or small population sizes, caused only by demographic stochasticity, occur on time scales on the order of hundreds of years. This observation further supports our prediction that environmental stochasticity generates much of the variance observed in the 17-year dataset we study here. It also calls into question the generality of conclusions about demographic stochasticity drawn from samples even decades long. If such forces strongly influence population dynamics of species with similar life histories, modeling will be required to correctly characterize the dynamics; studies relying solely on statistical assessments of data at hand are likely to miss significant dynamical processes.

### Environmental stochasticity

Our primary method for comparing simulation output to data focuses on comparing observed means and variances to estimates of those parameters’ sampling distributions derived by Monte Carlo simulation of the model. Within the portion of parameter space that admitted reasonable fits to the data, our simulations consistently matched the mean population size in the Bimini nursery; however, simulations regularly generated variances distinctly lower than that seen in the actual data set, even when data variance fell with the middle 95% of the sampling distribution of the variance. Specifically, simulations on average account for approximately 33-49% of the data variance. The variance in our models is generated entirely by demographic stochasticity and any instabilities caused by delayed breeding and age structure. What, then, caused the missing variance? We postulate it may have been a combination of variations in prey abundance, environmental stochasticity, including weather patterns and global climate change, habitat loss, and effects of fishing.

Whatever this environmental stochasticity is, we predict that its effects are relatively sparse, even though we underestimate the actual variance by a considerable amount. This prediction follows from a close inspection of the data from North Sound (Figure 1). It appears that the high and low points in this time series (2005 and 2008, respectively) may be “outliers”. Removing these points from the data set reduces the variance to 164.12, but leaves the mean at 75.13, both of which are in almost exact agreement with simulation mean and variance at default parameter settings. Therefore, the discrepancy between our simulations and the data appear to be driven by only two events in the 17-year data run. As stated above, we would hypothesize that these “outliers” are primarily driven by some type of environmental stochasticity.

The next step with this model is to incorporate environmental stochasticity so that more accurate assessments of population viability can be made [19]. However, such modifications will be difficult because stochastic environmental effects include an enormous array of possibilities. In addition, predator-prey dynamics, including juveniles as prey for both conspecifics and other species, should be modeled. Cannibalism, which has been documented in this species [50, 51], needs careful attention because it can have very drastic effects on population dynamics [52, 53].

## Conclusions

We used a stochastic, stage-structured model to identify the primary determinants of the mean and variance of juvenile lemon shark population size in Bimini. By including all demographic processes thought to be important in the population, we were able to capture the mean of the population size. However, we consistently underestimated the interannual variance. Because all the variables we included in the model were related to demographic processes, we would predict that this unaccounted variance is primarily driven by environmental processes. Our modeling approach is ideally suited to study populations where basic data on annual population size is available; all the technique requires are estimates from the data of mean population size and interannual variance from at least some age class or classes. However, even this requirement can be relaxed. In an inverse modeling scheme, any measurement made in the field that can be mapped to a variable in the model could be used to determine which combinations of parameters are a good fit [54]. The ability of this technique to predict bounds for parameters that are not easily estimated in the field has important implications for management and conservation, generating as it does predictions about which parameters and life cycle stages may be most sensitive to anthropogenic impacts such as overfishing or bycatch. For lemon sharks at Bimini, we show it is essential to include density-dependent mortality in the first age class and to incorporate delayed breeding to predict even basic population dynamics. We also show that adult lemon sharks must have a mortality rate below 0.17 in order for the population to remain viable. Although we have a relatively long data set (17 consecutive years), longer time series may be required to capture important, rare stochastic events [55]. These types of events, whether they be environmental or demographic, seem to be the primary factor in driving the fluctuations in the population size of juvenile sharks in Bimini.

## Methods

### Study site and field data

This study builds on field work conducted in Bimini Lagoon, Bimini, Bahamas (25°44N, 79°16W). The Biminis are located approximately 86 km east of Miami, Florida and provide habitat for numerous species of fish, arthropods, birds, and mollusks [56]. Of the three lemon shark nursery sites (as defined by [57]) in Bimini, our study focuses on the most northerly one, known as the North Sound. Between 1996 and 2012, standardized gillnet methods were used to capture juvenile lemon sharks within 45 days of parturition (Figure 1). These methods are assumed to be exhaustive, meaning that all individuals were caught in the study area each year. We use this assumption in our model. For a more detailed treatment of the gillnetting protocols and yearly censuses, see [33, 35, 58, 59]. All research was approved by the Bahamian Department of Marine Resources.

In addition to population censuses, genetic analyses from tissue samples were used to reconstruct family pedigrees [34, 35, 59] from which we estimate per-female annual fecundity in the Bimini population (Figure 3). Reproductive-age female lemon sharks (ages 12+) show strong philopatry to their natal nursery sites, with about 45% returning to a given nursery area to reproduce every other year [34]. Newborn and juvenile sharks (ages 0-2 years old) stay in these protected, mangrove-fringed nursery areas [50]. In addition, there appears to be very little dispersal among nursery sites in this region, so the population of juvenile lemon sharks in the North Sound is essentially closed [33]. At about age 3, lemon sharks enter their subadult phase (ages 3-11), begin to leave the lagoon area and move to deeper waters [50, 60, 61].

Our model is constructed to capture this natural history in such a way that key model parameters, such as mortality rates of different age groups, can be estimated from field data. This allows us to use inverse pattern-oriented methods to estimate other life history parameters that are otherwise difficult or presently impossible to measure directly.

### Model

We model the Bimini lemon shark population as an age-structured, Markov-chain stochastic process. We choose this formalism due to the complexity of the lemon shark’s life cycle—in particular the delay in breeding to the 12th year—and because breeding populations at nursery sites in any given year appear to be too small to be buffered from fluctuations due to demographic stochasticity. Since the maximum age for lemon sharks is thought to be 25 years [24, 28], we assume a maximum of 26 age classes (including the 0 ^th^ age class).

Let **x**(*n*) be the shark population vector at census time *n*; that is, its elements, *x*_*a*_(*n*), *a*∈{0,1,…,25}, *n*∈{0,1,2,…}, represent the number of lemon sharks of age *a* in the North Sound population, including all animals born to and breeding in the North Sound nursery, whether they are in the nursery or open ocean at census *n*. Age class 0 represents sharks born the year of the census. To match the timing of the actual Bimini census, we assume that this census occurs just after reproduction (i.e., pups are born April/early May and are sampled late May/June).

#### Fecundity

We assume an equal sex ratio and that females only reproduce every other year after their 11th year of life [34]. The equal sex ratio assumes equal numbers of females and males born and equality in the survival rates of both sexes throughout their lives. Let *R* be a random variable taking on values in {0,1,2,…} with probability density {*p*_0_,*p*_1_,*p*_2_,…}. We interpret *R* as the number of offspring born to a particular breeding female, and *p*_*i*_ as the probability that a female gives birth to *i* pups. Let the number of breeding females in year *n* be denoted *b*_*n*_; that is,
1

the coefficient of 1/4 follows from the assumptions of equal sex ratio and biennial breeding with the further assumption that, for each age class, the proportions of females breeding in even and odd numbered years are equal. We assume that all breeding females have the same reproductive potential regardless of age class, time or population density. Therefore, the set {*R*_*i*_(*n*);*i*∈{1,2,…,*b*_*n*_}} is a collection of independent, identically distributed random variables, and *R*_*i*_(*n*) is the reproductive output of the *i*th female in year *n*. Therefore,
2

is the total reproductive output of the population in year *n*. Note that the dependence of *B* on *n* comes only through the number of breeding females in year *n*, not through *R*(*n*).

The probability density, {*p*_0_,*p*_1_,*p*_2_,…}, for *R*(*n*) can be obtained from a variety of assumptions. We consider two possibilities. In some simulations, we obtain this density from data; in particular, each *p*_*i*_ is set to the observed frequency of females producing *i* pups (Figure 3), with the convention that *p*_*j*_=0 for all *j*>18. In the second case, we assume that all *R*_*i*_(*n*)’s are Poisson-distributed with fixed mean *λ*. In this case, the probability density for the total population fecundity in year *n* becomes
3

A Poisson distribution is often assumed to be a good fit for a birth process. We explicitly test that assumption here.

#### Mortality

We assume that the probability of mortality is evenly distributed across all individuals in a given age class; therefore, within an age class the number of sharks that die between censuses is distributed binomially. Generally speaking, the parameter of that distribution—the probability that a given shark dies—could potentially depend on population size. However, in the case of lemon sharks, we only have evidence for density-dependent mortality in the first age class [28, 33]. There is insufficient evidence to support either density-dependent or -independent mortality assumptions in other age classes; indeed, very little is known about lemon sharks once they leave their nursery area. Therefore, as a first approximation we chose density-independent mortality for all age-classes above the first.

In this first age class, the probability that a shark pup dies between birth (age class 0) and its second census (i.e., dies in age class 1) is a generally increasing function of the size of its cohort (*x*_0_(*n*)) in the lagoon in that year [28, 33]. This type of density-dependent mortality may be a result of reduced prey resources (although the population does not appear to be prey-limited in any way), predation from large barracudas, predation from other shark species, or cannibalism, which has been documented for this population [50, 51]. We model this cohort-density dependence with a generalized Michaelis-Menten function (equivalent to a Hill function):
4

with (constant) Hill and shape parameters *h*≥1 and *k*>0, respectively. Let *M*_0_(*n*) be a random variable representing the number of sharks born in year *n* that die between their first and second censuses. Then *M*_0_(*n*) has probability distribution
5

where we suppress the notation the dependence of  on *x*_0_(*n*) for clarity.

As a first approximation, we assume that no age classes except the first have density dependent mortality. We further assume that the probability of mortality for any shark in age classes 1 or higher is invariant across individuals regardless of age (this assumption could be relaxed with our model structure). We denote this constant probability as *μ* and define *M*_*a*_(*n*) to be a random variable representing the number of deaths in age class *a*∈{1,2,…,25}. Then
6

It is important to note that many of these assumptions can be relaxed without altering the form of our model (see below). For example, here we assume no fishing mortality because there is no shark fishery in Bimini. One could easily incorporate such an assumption into *μ*, and even make *μ* age-class- and (or) density-dependent with fairly obvious alterations to the probability distribution for mortality, equation (6), which have no effect on the overall model form.

#### Model form and parameterization

The development above generates a model with the following form:
7

where **x**_0_ is the initial age distribution.

Application of model (7) to the lemon shark population requires field estimates of fecundity and mortality. Starting with the former, as noted above we can estimate the probability distribution directly from data (Figure 3), or we can assume that per-female reproductive output is Poisson-distributed with mean *λ*. Data from Bimini over the last 20 years suggests that *λ*≈6.1 pups per female [34], although this value is somewhat lower than that used in previous modeling studies (perhaps because of the high mortality of pups between birth and our sampling season; in this case the 6.1 pups per female simply represents the number of sharks that make it past that interim period) [23, 28].

Less is known about mortality in this species. The first-year mortality function, equation (4), requires two parameters: the Hill parameter (*h*) and the shape parameter (*k*), whereas non-first-year mortality only requires an estimate of mean per-shark probability of mortality, *μ*. Because of the lack of data, we compare model output to population data from the Bimini study to define a range of potential values for these parameters using a sensitivity analysis similar to that in [54]. We describe this method in the next section.

### Simulations and analysis

For clarity of exposition we will refer to sharks from ages 0 to 2 as juveniles, from ages 3 to 11 as subadults and above age 11 as adults. Juveniles, as defined above, are the animals actually caught each year in the Bimini nursery census. We evaluate the model by comparing its behavior to the Bimini nursery census data. Model (7) was implemented and all analyses were conducted using the open-source computing language R [62].

We compared observed means and variances to estimates of the sampling distributions for mean and variance generated by simulations of the model. To construct the sampling distribution for a single parameter set and sample of length *n*, we ran 100 simulations of the model and obtained a sample of *n* contiguous time steps from each of the 100 runs. The mean and variance was then calculated from each sample to give distributions of 100 means and variances of size *n* for a particular simulation scenario. These distributions are our estimates of the sampling distributions for the mean and variance. In a given simulation, if *n*≤200, then the simulation was run for 300 time steps (or until the population went extinct). The first 100 time steps were removed, and the sample was taken from a (pseudo)randomly chosen interval of size *n* from the remaining interval of 200 time steps. If *n*>200, then the simulation was run for 100+*n* time steps, the first 100 being eliminated and the sample mean and variance taken from the remaining *n* time steps.

This Monte Carlo technique to estimate sampling distributions allowed us to use an inverse pattern-oriented technique to quantitatively compare simulations and data [54, 63–65]. With this approach, we evaluate where the actual means and variances of population size from field data would fit in the simulation sampling distributions over variations in parameter values and “study lengths” (sample sizes). In the first case, we explored 9000 distinct parameter combinations (*λ*: range 1-15 (step size = 1); *k*: range 10-200 (step size = 10); *μ*: range 0.01-0.30 (step size = 0.01); *h* fixed at 1). Note that, although *λ* is estimated directly from data (Figure 3), it is still of interest to examine a range of values for *λ* to evaluate the type of compensatory responses generated by variations in *λ*. We fixed *h*=1 in equation (4) because this gave the best fit (least sum of a squares) to the relationship between mortality and density obtained by [28]. Likewise, we also limited *k* below 200 because larger values produce a linear mortality rate that greatly underestimates that measured by [28] and [33].

To assess how well any given parameter combination represented the field data, we determined if both the mean and variance of the field data set fell within the middle 95% of the estimated sampling distribution (i.e., removing the extreme 2.5% of low and 2.5% of high values) of means and variances generated from the simulations of a particular parameter combination. This amounts to a 2-tailed test against the model’s parametric distribution with *α*=0.05. The fit between model and data was deemed “good” if (i) mean size of the juvenile population size from the data fell within the middle 95% of the distribution of mean juvenile population sizes from the simulations; (ii) variance in juvenile population size from the data fell within the middle 95% of its distribution from the simulations; and (iii) the population remained extant after 300 years in each of the 100 runs. By eliminating parameter combinations that fail to satisfy any of these three criteria, we effectively constrain possible values for the unknown parameters (see Figure 2).

#### Testing the effect of sampling length

In our initial analysis, we generated sampling distributions for the mean and variance for samples of size 17 using the techniques outlined above; that is, we sampled a randomly-chosen sequence of 17 consecutive years—after initial transient dynamics have settled down—from each simulation as an analogue to the 17 consecutive years of field data at hand. However, a question arises regarding how well a 17 year data set represents centuries of ecological dynamics [55]. To assess this, we compared field data to sampling distributions of various lengths of simulation time series from 10 to 400 years (Figure 5). The procedures used to derive the sampling distributions of the variance for these various sample lengths was identical to that described above.

## Reviewers’ comments

### Reviewer report 1

Dr. Yang Kuang (School of Mathematics and Statistical Sciences, Arizona State University)

This paper deals with the task of modeling the lemon shark population based on a longitudinal data covering 17 years. The model is a hybrid discrete model with stochastic component, It provides not only a way to fit data sets that are intrinsically stochastic, it also present a way to quantify the variance of stochasticity. It also covered the background material and modeling efforts in other model forms nicely. Nevertheless, I have a couple of minor comments/suggestions.

1. The model equation (7) has a typo in the equation of *x*_*a*_(*n*+1), which shall be *x*_*a*−1_(*n*)−*M*_*a*−1_(*n*).

*Authors’ response: We thank the reviewers (both Dr. Kuang and Dr. Jacob) for catching this typo. We have corrected it.*2. Please either provide a table listing the values of the census data, or label them near the circles in Figure 1.

*Authors’ response: We invite readers interested in obtaining the data to send an email to the corresponding author.*

3. Can you explain why you did not try the Leslie matrix model to fit the data?

*Authors’ response: We agree that the model can be expressed in using a matrix formalism. Our attention was focused primarily on defining a stochastic process that could be implemented and analyzed computationally. Since it is a relatively straightforward model, we were satisfied with the “brute force” motivation given. However, we agree that a matrix formulation has advantages vis á vis a more analytical attack on both dynamics (speaking also to reviewer 2’s (Dr. Jacob’s) comments) and the inverse problem. The parameter estimation technique we employ here, however, is indifferent to the model formalism.*

Dr. Christine Jacob (Applied Mathematics and Informatic unit (INRA), France)

1. Abstract and general comments

The paper deals with the modeling of the population dynamics of lemon sharks at Bimini, Bahamas, from 1996 to 2012 by a (stochastic) time-homogeneous (i.e. constant environment) and aged-structured branching process with a population dependent mortality for the juveniles aged less than 1 year. The population of females able to have offspring is assumed to be a fixed part of the animals aged from 12 to 25 years. The model depends on unknown fecundity and mortality parameters. The fecundity distribution may either be estimated by the empirical histogram derived from literature or by a distribution assumed to be Poisson(*λ*). The parameter *λ* and the mortality parameters are estimated by a range of values that are validated by a kind of sensitivity analysis given in the literature (“inverse pattern-oriented technique”): the empirical mean and variance for each of the 9000 different processes corresponding to a particular parameters combination are compared to the observed empirical mean and variance (the criteria is described in “Methods (Simulations and analysis)”).

*Authors’ response: We thank the reviewer for her accurate synopsis of our main points. We would like to make a clarification here, however. The fecundity data were obtained in the field under the direction of one of us (SHG) while another of us (ERW) participated in some of the field work. This paper presents the most up-to-date data set which, at the time of publication, has not been entirely published elsewhere.*

According to the authors, this is the first stochastic model built for this population dynamics. Taking the variability of the dynamics is an important issue.

The authors concluded that the simulated variance explains only around one third of the observed variance. The authors notice that the presence of the two observed outliers (2005 and 2008) is sufficient to explain this difference and that these outliers could be explained by some important deviation from a constant environment. Hence my questions: how to explain the extremal events (does the hurricane Katrina capable to explain the important population size of 2005?), what about other factors such as possible random errors of observation (under or overestimation)? What about a validation of the model based on other sets of observations?

*Authors’ response: It is unclear what environmental factors may be driving the population dynamics in 2005 and 2008. The possibility that part of the explanation may involve Tropical Storm/Hurricane Katrina (which became a hurricane just before making its first landfall in Florida, after the center had passed the Bahamas) is very intriguing. Indeed, there were more hurricanes than average (per year) between 2003 and 2005. Unfortunately, we have no detailed understanding (from evidence) how hurricane activity affects reproduction or survival of lemon sharks in the Bimini lagoon, in particular how it could cause the nursery population to spike high in 2005 (or low 3 years later). So really all we could do with this interesting hypothesis at this time is report coincidences. There are also a number of competing hypothesis of the cause of environmental effects, and we propose several others in the discussion. But again, our main point is not to identify the exact cause of what we claim is unexplained variance, just that some of the variance is unexplained by demographic stochasticity, and the likely culprit is environmental stochasticity.*

*As for error in estimation (see also Dr. Hyrien’s comments below), the field techniques used to count sharks in the nursery are exhaustive or nearly so (Gruber et al. 2001), so we assume no under- or overestimation of the population size for the early age classes. We clarify this point in the revised manuscript.*

*As for validating with another set of data, we completely agree, but argue that another model would be required for validation (see below). At the moment, this is all the data at hand, so further validation will require more time and a great deal more effort, which is already nontrivial, in the field.*

The model validation is based only on simulations. From a probabilistic point of view, knowledge of the branching process could help. For example, the behaviour of the process, in particular its mortality, depends not only on *μ* but also on . From a statistical point of view, the “estimation” of the parameters is done first by choosing a rough range of values containing, for each parameter, its estimation derived from the litterature, *h* being set to 1, which leads to 9000 different combinations of the parameters, and second by refining this range of values by comparing the empirical mean and variance derived from each of the 9000 models with the empirical mean and variance derived from the observations.

*Authors’ response: We completely agree. However, this brings up an important point that we clarify in the manuscript. Our bounds on the parameters apply only to demographic variation, not environmental. In other words, we offer bounds on births, deaths and density-dependence during “normal” years. However, from the original manuscript, “it is important to note that even for parameter combinations that qualified as good fits, all greatly underestimated annual variance observed in the actual data”, because we claim that the model does not capture relatively rare environmental events. Therefore, we already expect that the model will not pass (strict) validation. We return to and extend this point in the next comment.*

The authors should justify this approach compared to other methods such as Bayesian analysis. This is an important point for publication.

*Authors’ response: We realized after reading the reviewers’ comments that our rhetoric went beyond our analysis. In fact, the Monte Carlo technique we employ does not “parameterize” a model per se. Instead, it systematically samples parameter space to clarify bounds for the parameters. It does not attempt to attach a probability or likelihood to a parameter value given the data as likelihood and Bayesian techniques would, nor does it attempt to optimize a formal fitting function as would a genetic algorithm. We chose the technique we did out of a desire to be conservative. Given the very limited information about lemon sharks, especially demographics outside the nursery, we have no model for variance, covariance and (or) likelihood functions required by these other techniques. Instead of adding another layer of uncertainty, we chose this more direct, albeit weaker, technique. Another important point is that, strictly speaking, these parameter bounds are obtained under the assumption of time-homogeneity of the stochastic process, an assumption we argue is violated. That there appear only to be 2 outlier years, and that we can match means and variances reasonably well (although always underestimating the latter) suggests our bounds are probably close, but perhaps biased. So, we see our model as a first approximation to the dynamics driven by demographics, and the bounds derived for its parameters as a resource that can be used to define a reasonable prior distribution for a subsequent Bayesian analysis on an independent data set from this or a related population.*

Note also that the mean and variance of each data set has some meaning only if the underlying process is stationary until its extinction, which is not guaranteed for all parameter combinations.

*Authors’ response: We agree, and addressed it the original manuscript by fit criterion 3), “the population remained extant after 300 years in each of the 100 runs”. Although we lack proof that the process is stationary, this criteria effectively eliminates parameter combinations for which the process is non-stationary, since in those cases the process tends to run to the absorbing state (extinction) within 300 time steps in at least one of the 100 runs.*

The model described by (7) (“Model form and parameterization”) presents a crucial error: “ *x*_*a*_(*n*+1)=1−*M*_*a*−1_(*n*)” is a null or negative quantity! I hope that this is only a typographical error…(this error is also in the preprint found on internet). This is a crucial point for publication.

*Authors’ response: It’s indeed a typo, which we have corrected. See our response to Dr. Kuang’s comments.*

2. Minor issues not for publication
1. In the abstract and the “Background”, the authors start by pointing out the role of the anthropogenic mortality on the population while the study concerns only the natural mortality.

*Authors’ response: Anthropogenic mortality is important for many species of sharks and stingrays, but no known anthropogenic mortality currently affects lemon sharks at our study site. This makes this population interesting as a test site for other species since we can examine dynamics of a population that is not currently experiencing fishing pressure, but has a natural history similar to many species that are.*

2. In “Background”, write “2012” instead of “present”.
3. In “Background” further, some quantities are described beyond the location of their first appearance, for example “the pattern oriented techniques” are described only in the further section “Methods (Simulations and analysis)”, and the parameters *λ*, *μ*, *k*, etc, are described in the further section “Methods (Model)”.
4. In “Background” write “Poisson” instead of “Possion”.

*Authors’ response: These points have been addressed in the revised manuscript.*5. In “Background” further: Figure 5 is a bit strange and not clear. In the same figure, the observed variance and simulated box plots are represented (the scale of these quantities differ). I would prefer simulated variances instead of the box plots. Moreover the authors deduce that the simulated variances tend to increase towards the observed variance as the sample size increases while I only see that the median (or the mean?) is increasing.

*Authors’ response: In Figure*5*, only variances are plotted. Therefore, they are all on the same scale. The box plots represent the distribution of variances obtained from 100 simulation runs from each of which a sample of size “Sampling Length” (indicated on the horizontal axis) has been taken. (In essence, each box is a Monte Carlo estimation of the sampling distribution of the variance for samples of size “Sampling Length”.) For example, consider the box for “Sampling Length” 400 (the right-most box in the figure). To construct that box, we start with a single simulation of length 500 time points. We then threw out the first 100 time points and calculated the variance of the remaining 400. We repeated this procedure 99 times to obtain 100 variances. The box plot shows the distribution of these 100 variances (median variance with quartiles and outliers greater than 1.5 times the inner quartile range from the edge of the box). We recognize that we did not make our main argument clear enough. So, because Figure*5*is central to our argument, we reiterate our thinking here and have tried to clarify it in the main text as well. What we see in Figure*5*is an increase in the median of the variances as the length of the “study” increases. However, this median, indeed the inner quartile range, never reaches the observed variance even for samples of 400 years. For samples of 20 years, nearly the length of the field study, not one of the 100 simulations–not even an outlier–generated a variance equal to or greater than our observed variance (although 2 of 100 did for 10 year samples–see Figure*5*). If we lump the two sampling distributions of the variance for sample sizes nearest ours (10 and 20), then the observed variance was exceeded in only 2 of 200 simulation runs, suggesting a p-value no larger than 0.01. So, the demographic model can generate, on occasion, variances that approach or exceed what we observe in the field, but only with a reasonable probability for studies of at least 100 years, and even in the best case (a 400 year study) it would be never more than 25% or so (being conservative). Therefore, the variance from our sample of 17 years is unlikely to have been generated by demographic stochasticity. It is also unlikely to have been generated by complex dynamics (which would also have been captured in the model and is anyway unlikely with such limited density-dependence). It is also unlikely to be generated by the assumption of equal sex ratios (see response to Dr. Hyrien’s comments). Therefore, we are left with a diagnosis by exclusion—the most likely candidate, in our opinion, for the higher than predicted variance is environmental stochasticity. This conclusion is further supported by the apparent outliers in 2005 and 2008.*

*And this leads to our take-home message—a 17 year time series is long for a field study, but small in absolute terms when taken from a population that exists thousands of years at least. Yet even in this tiny sample, we find significant environmental effects. This observation, along with many others from longer field studies, we think points to the importance of discussions regarding the usefulness of demographic models for real field populations and the timeliness of tackling the daunting problem of modeling environmental forcing.*

6. In “Discussion (Mortality assumptions)”, replace “population-dependent” by “non-stationary”.
7. Please do not put capital letters when uncessary (ex: “Study Site and Field Data” should be replaced by “Study site and field data”.
8. In “Methods (Mortality)”: “where we suppress the dependence of …” should be replaced by “where we suppress in the notation the dependence of …
9. In “Methods (Simulations and analysis)”: explain what means “the middle 0.95 of the distribution”.

*Authors’ response: These points have all been addressed in the manuscript.*

Dr. Ollivier Hyrien (Department of Biostatistics and Computational Biology, University of Rochester)

This manuscript presents a model of the dynamics of the population of juvenile sharks in the Bimini nursery in the Bahamas. Previous demographic models of such populations have considered age-structure and density dependent mathematical models. All of them were deterministic however, and this manuscript considers an age-structure, stochastic model formulated as a discrete time Markov chain.

The primary objective of the study is to investigate the variability in the number of juvenile sharks observed in the lagoon every year using the proposed stochastic model. The main conclusion of the paper is that the observed number of juvenile sharks, as estimated during census, varies considerably more than when predicted by the proposed model, and that the extra variability can be attributed to environmental factors.

In my opinion, there is not enough evidence that such a conclusion can be reached based on the data presented in the manuscript.

The variance is estimated as ≈498 sharks2 per year based on data collected during yearly census. This estimate is obtained based on 17 observations. By visually extracting the values of the data from the plot, I find that removal of the observation from 2005 decreases the variance to a value slightly above 200 sharks2, corresponding to more than a 50% reduction in the count. The authors can do exact calculations using the actual values, but the point made here is that the 2005 observation has a high influence on the point estimate for the variance of the number of juvenile sharks in the lagoon per year. Thus, the value of 498 for the variance may be an overestimation of the actual value. At the least, the estimate should not be used without a properly constructed confidence interval, which may be quite wide since only 17 observations were available.

*Authors’ response: We completely agree that removal of the data from 2005 decreases the variance considerably (as does removal of 2008). Hence, we wrote in the original manuscript (and have left untouched in the revision), “It appears that the high and low points in this time series (2005 and 2008, respectively) may be outliers. Removing these points from the data set reduces s2 to 164.12 but leaves the mean at 75.13, both of which are in almost exact agreement with simulation mean and variance at default parameter settings.” We disagree that the observed value is an overestimation. We did not sufficiently emphasize the exhaustive nature of the sampling (see above responses to comments, manuscript and supporting literature). We are really comparing the model prediction to an exhaustive census. From our explanation of Figure*5*above and the Monte Carlo analysis we performed, we conclude that the model has a low probability (twice in 200 runs for samples sizes bracketing ours (10 and 20)—see response to comments above) of generating the observed census variance.*The authors performed numerical simulations of the population dynamics using their model. A summary of the simulations is presented in Figure 5. The graph includes boxplots of the 100 simulated values available each year.

*Authors’ response: This is incorrect, as we explain in our response to Dr. Jacob’s comments. The box plots are distributions of variances. Again, we attempt to make the construction of Figure*5*more clear in the revision.*

As discussed in the manuscript, the plot suggests clearly an increase in the variance of the number of sharks as time increases (time 0 seems to be the time of origin of the simulation). There is no attempt at explaining why the sample variance increased with the number of years elapsed since the starts of the simulations. A plausible explanations is that the initial conditions used to initiate the Markov chain differs with the stationary distribution of the chain, assuming that such a distribution exists. There does not appear to be any description of how the initial values of the process were specified.

*Authors’ response: Again, we apologize for not making our methodology sufficiently clear. In each simulation, the initial conditions were always the same, and were chosen to represent the best field estimates. Then each simulation was run for at least 300 time points, and the first 100 time points were removed to eliminate transients and allow the process to settle into, or close to, its asymptotic dynamics. We explain this more clearly in the revision.*

*In the original manuscript, we did in fact provide an explanation of the phenomenon. From the original discussion: “Apparently, random walks to exceptionally large or small population sizes, caused only by demographic stochasticity, occur on time scales on the order of hundreds of years. This observation further supports our prediction that environmental stochasticity generates much of the variance observed in the 17-year dataset we study here”.*

Also, why not use the variance at t = 400 instead of t = 17 to make a comparison with the observed point estimate of the variance computed from the real data (498)? The value from the simulations seems to approach, if not exceed, 400, which is much closer to 498 than the value of 176 used in the discussion. It is unclear whether the variance of the process has approached its limiting value by time t = 400. Various techniques exist to study the asymptotic distribution of Markov chains. They could be used to derive the true value of the asymptotic variance of the process.

*Authors’ response: Again, this is a misinterpretation of what we did, caused by our lack of clarity about Figure*5*. Our response to Dr. Jacob’s comments should clear this up. One further clarification is in order here: for samples of length 17 years, we performed 100 simulations. In each, we ran the simulation for 300 years, removed the first 100 time points (as described above) and then chose a continuous 17 year interval (pseudo)randomly from the remaining 200 time points. Since this was done for each of the 100 repeated simulations, we obtained 100 variances to generate our estimate of the sampling distribution of variances for samples of 17 years from the model. It’s this distribution to which we compare the actual data.*

As a side note, 100 runs appear rather small to study a stochastic system via simulations. Were the simulations very time consuming to run?

*Authors’ response: Yes, the simulations were time consuming so we only conducted 100 runs of (at least) 300 time points for each of the 9,000 parameter combinations and all the various sampling lengths shown in Figure*5*.*The model uses a Poisson distribution to describe the number of pups per female (presumably per year). The fitted distribution and the empirical histogram are plotted together in Figure 2. Unlike stated in the manuscript, the model does not provide an acceptable fit to the data. At least, there is no p-value from a goodness-of-fit test provided to support the choice of this model. On page 5 (top), the authors state that the Poisson assumption increased the variance of the model (which I interpret as “increasing the variance of the number of juvenile per year”) compared to the model that uses the empirical distribution plotted in Figure 2. Since the empirical distribution is likely less biased than the Poisson distribution, why not use it? The values from the simulations based on the empirical distribution were not reported. They should be included in the manuscript.

*Authors’ response: We wanted to test the Poisson assumption against the empirical distribution of births, because many field studies do not have access to an empirical distribution of births. We say the model provides an acceptable fit to the data, because there are several parameter combinations (for both the Poisson and empirical distributions) that produced distributions for the mean and variance of population size from the simulations that contained the field estimates of mean and variance.*

The estimation procedure is barely described in the manuscript. There exists a vast literature devoted to estimation for discrete time Markov chains, but no justification/discussion is given about why the authors used an inverse pattern-oriented technique.

*Authors’ response: This is substantially the same point Dr. Jacob brought up, and we deal with it at length in response to her comments.*

The variance in the simulation may also be underestimated by the assumption of an equal sex ratio. What does this assumption really mean? Is it per litter or is it per year? How does it work with the assumption that the number of pups per female is Poisson (and thus odds number of pups may occur)? For example, does it mean that the numbers of pups per female were not mutually independent (adjustment were needed to enforce the assumption)? Was this assumption absolutely needed (since the authors are using a simulation model which can be relatively easily adapted)?

*Authors’ response: The assumption of an equal sex ratio in our study means an equal sex ratio in litters and equal survival of males and females. This assumption is supported on both empirical and theoretical grounds. It doesn’t matter if an odd number of pups occurred in the litter, because we don’t distinguish the age classes by sex. We simply assume that the entire adult population, divided by two, should equal the number of potential reproducing females. This assumption was not strictly needed, but we weren’t interested in the effect of males versus females, so it was a simpler model. But this assumption is not the cause of the underestimated variance since the population model includes both males and females.*Finally, there is no precise explanation about how the data were collected. A description of the sampling process would be useful for the readers to determine whether the values presented in Figure 1 are actual (exact) counts obtained from exhaustive sampling of the nursery or whether they correspond to estimates of the actual counts. In the latter case, the point estimate of the variance (498) is a biased estimator, which would overestimate the actual variance since it combines both the sampling error and the biological variability of the number of sharks present in the nursery in any year.

*Authors’ response: We agree that the details of the sampling will clarify its exhaustive nature. However, a detailed description already exists in well-cited literature, and indeed the study is well-known among fisheries biologists. Elucidation of the field methods here would be a bit redundant and outside the scope of this paper, which focuses on the model.*

On page 14, the probability of *M*_0_(*n*)=*m* should be conditioned on *x*_0_(*n*).

*Authors’ response: We agree and have made this correction.*

As noted above, the main conclusion made by the authors is that the difference in variance may be due to environmental factors. While this statement may be true (as such factors do play a role in ecological systems), it is not proven by the proposed study.

*Authors’ response: We agree that we have not proven anything, nor were we trying to. We do, however, continue to defend our main point–it is unreasonable to conclude, based on the extensive Monte Carlo methods we employ, that the model sufficiently explains the observed variance in the field. It is, however, reasonable to conclude the converse. As we argue in response to Dr. Jacob’s comments, we suggest that the model fails to capture this variance because it is missing something vital. What’s missing is not complex dynamics. We are left with environmental stochasticity, or an incorrect simplifying model assumption as potential causes. But, since it appears that two (maybe only one) year is generating the variance that the model cannot explain (and the model explains the remaining years well), we suggest environmental stochasticity as the main cause.*

I would also recommend the author to place the description of the model in the Results section. In the present version, there are parameters that appear in the results and discussion section that are not defined before (e.g., half saturation value).

Change the label of Figure 2 and Figure 3 since the latter seems to be cited before the former in the text.

*Authors’ response: We agree and have made these changes. We thank all three reviewers for their detailed and insightful comments. They have helped us greatly in focusing our argument.*

### Reviewer report 2

Dr. Yang Kuang (School of Mathematics and Statistical Sciences, Arizona State University)

The revised version is acceptable now.

*Authors’ response: We appreciated your comments on the first round of reviews.*

Dr. Christine Jacob (Applied Mathematics and Informatic unit (INRA), France)Authors need to clarify the assumption of stationarity before extinction, both for the data and for the model. Since the model can be written as a BGW multi type branching process whose types are the different age classes, then the natural property for a BGW branching process corresponding to some stability is criticality. For a critical BGW process, the corresponding Q process (process conditioned on the non-extinction in the distant future) is stationary. This criticality corresponds to the subset of parameter space such that the Perron’s root p of the mean matrix of the process is equal to 1. So in order to improve the simulations, that is in order to remove this growth trend in the simulated variances in Figure 5, it would be really worthwhile to restrict the parameters estimates of the model to those belonging to this subset. Otherwise the methodology is not accurate.

*Authors’ response: We completely agree with the reviewer here. However, the numerics clearly supported stationarity in the cases satisfying our “fit” criteria described in the methods, so we have a high degree of confidence in the result.*

1. p. 4, line 13, what means “field estimates”

*Authors’ response: Here “field estimates” mean the estimate of the population mean and variance from the yearly censuses. In the revised manuscript, we have included “(census)” to make this point more clear.*

2. p. 12 in paragraph Model, there are 2 different notations for *x*_*a*_(*n*), moreover x(n) and and “n” should not be typo

*Authors’ response: These typos have been fixed.*

3. p. 13 *R*_*i*_ should be written *R*_*i*_(*n*). The same for R

*Authors’ response: These typos have been fixed.*

4. write (*x*_0_(*n*))^*h*^ instead of *x*_0_(*n*)^*h*^

*Authors’ response: We have now made this change.*

5. p. 14, last formula, write *P*({*M*_0_(*n*)=*m*}|*x*_0_(*n*)) or *P*(*M*_0_(*n*)=*m*|*x*_0_(*n*)) instead of *P*({*M*_0_(*n*)=*m*|*x*_0_(*n*)})

*Authors’ response: We have now made this change.*

6. p. 15 line 6, “the number of deaths”

*Authors’ response: We have now made this change.*7. p. 27 the of Figure 2 correspond to Figure 3. Define half saturation value in Figure 4 legend.

*Authors’ response: We have adjusted the Figure*4*legend, but do not see a problem with the Figures*2*and*3*legends.*

## References

[1] Baum JK, Myers RA, Kehler DG, Worm B, Harley SJ, Doherty PA (2003). **Collapse and conservation of shark populations in the Northwest Atlantic**. Science.

[2] Lewison RL, Crowder LB, Read AJ, Freeman SA (2004). **Understanding impacts of fisheries bycatch on marine megafauna**. TRENDS Ecol Evol.

[3] Hutchings JA, Myers RA, García VB, Lucifora LO, Kuparinen A (2012). **Life-history correlates of extinction risk and recovery potential**. Ecol Appl.

[4] Senko J, White ER, Heppell SS, Gerber LR (2014). **Comparing bycatch mitigation strategies for vulnerable marine megafauna**. Animal Conserv.

[5] Myers RA, Baum JK, Shepherd TD, Powers SP, Peterson CH (2007). **Cascading effects of the loss of apex predatory sharks from a coastal ocean**. Science.

[6] Heithaus MR, Frid A, Wirsing AJ, Worm B (2008). **Predicting ecological consequences of marine top predator declines**. Trends Ecol Evol.

[7] Baum JK, Worm B (2009). **Cascading top-down effects of changing oceanic predator abundances**. J Animal Ecol.

[8] Ferretti F, Worm B, Britten GL, Heithaus MR, Lotze HK (2010). **Patterns and ecosystem consequences of shark declines in the ocean**. Ecol Lett.

[9] Heithaus MR, Frid A, Vaudo JJ, Worm B, Wirsing AJ, Carrier JC, Musick JA, Heithaus MR (2010). **Unraveling the ecological importance of elasmobranchs**. Sharks and Their Relatives, Volume II.

[10] Heupel MR, Knip DM, Simpfendorfer CA, Dulvy NK (2014). **Sizing up the ecological role of sharks as predators**. Mar Ecol Prog Ser.

[11] Kyne PM, Carlson JK, Ebert DA, Fordham SV, Bizzarro JJ, Graham RT, Kulka DW, Tewes EE, Harrison LR, Dulvy NK: **The conservation status of North American, Central American, and Caribbean Chondrichthyans****.** Tech. rep., IUCN Species Survival Commission Shark Specialist Group, Vancouver, Canada 2012

[12] Worm B, Davis B, Kettemer L, Ward-Paige CA, Chapman D, Heithaus MR, Kessel ST, Gruber SH (2013). **Global catches, exploitation rates, and rebuilding options for sharks**. Mar Policy.

[13] Caswell H (2001). Matrix Population Models: Construction, Analysis, and Interpretation.

[14] Brauer F, Castillo-Chávez C (2012). Mathematical models in population biology and epidemiology. Texts in Applied Mathematics.

[15] Gourley SA, Kuang Y (2004). **A stage structured predator-prey model and its dependence on maturation delay and death rate**. J Math Biol.

[16] Wang H, Nagy JD, Gilg O, Kuang Y (2009). **The roles of predator maturation delay and functional response in determining the periodicity of predator–prey cycles**. Math Biosci.

[17] Neubert MG, Caswell H (2000). **Density-dependent vital rates and their population dynamic consequences**. J Math Biol.

[18] Morris WF, Doak DF (2002). Quantitative conservation biology: theory and practice of population viability analysis.

[19] Ovaskainen O, Meerson B (2010). **Stochastic models of population extinction**. Trends Ecol Evol.

[20] Jenouvrier S, Holland M, Strœve J, Barbraud C, Weimerskirch H, Serreze M, Caswell H (2012). **Effects of climate change on an emperor penguin population: analysis of coupled demographic and climate models**. Global Change Biol.

[21] Mills LS (2012). Conservation of wildlife populations: demography, genetics, and management.

[22] McCarthy MA, Possingham HP (2012). Encyclopedia of Environmetrics.

[23] Hoenig JM, Gruber SH (1990). **Life-history patterns in the elasmobranchs: implications for fisheries management**. NOAA Tech Rep.

[24] Cortés E (1998). **Demographic analysis as an aid in shark stock assessment and management**. Fisheries Res.

[25] Mollet H, Cailliet G (2002). **Comparative population demography of elasmobranchs using life history tables, Leslie matrices and stage-based matrix models**. Mar Freshwater Res.

[26] Cortés E (2002). **Incorporating uncertainty into demographic modeling: application to shark populations and their conservation**. Conserv Biol.

[27] Beerkircher L, Shivji MS, Cortés E (2002). **A Monte Carlo demographic analysis of the silky shark (*****Carcharhinus falciformis*****): implications of gear selectivity**. Fisheries Bull.

[28] Gedamke T, Hoenig JM, Musick JA, DuPaul WD, Gruber SH (2007). **Using demographic models to determine intrinsic rate of increase and sustainable fishing for elasmobranchs: pitfalls, advances, and applications**. North Am J Fisheries Manag.

[29] Tsai WP, Liu KM, Joung SJ (2010). **Demographic analysis of the pelagic thresher shark,*****Alopias pelagicus*****, in the northwestern Pacific using a stochastic stage-based model**. Mar Freshwater Res.

[30] Dulvy NK, Forrest RE, Carrier JC, Musick JA, Heithaus MR (2010). **Life histories, population dynamics, and extinction risks in chondrichthyans**. Sharks and Their Relatives, Volume II.

[31] Cortés E (2007). **Chondrichthyan demographic modeling: an essay on its use, abuse and future**. Mar Freshwater Res.

[32] McCauley DJ, Mclean KA, Bauer J, Young HS, Micheli F (2012). **Evaluating the performance of methods for estimating the abundance of rapidly declining coastal shark populations**. Ecol Appl.

[33] Gruber SH, de Marignac JR, Hoenig JM (2001). **Survival of juvenile lemon sharks at Bimini, Bahamas, estimated by mark-depletion experiments**. Trans Am Fisheries Soc.

[34] Feldheim KA, Gruber SH, Ashley MV (2002). **The breeding biology of lemon sharks at a tropical nursery lagoon**. Proc R Soc.

[35] Feldheim KA, Gruber SH, Ashley MV (2004). **Reconstruction of parental micro satellite genotypes reveals female polyandry and philopatry in the lemon shark,*****Negaprion brevirostris***. Evolution.

[36] Brown CA, Gruber SH (1988). **Age assessment of the lemon shark,*****negaprion brevirostris*****, using tetracycline validated vertebral centra**. Copeia.

[37] Kessel ST: **An investigation into the behavior and population dynamics of the lemon shark (*****Negaprion brevirostris*****)****.** *Ph.d. diss.*, Cardiff University: United Kingdom; 2010

[38] Pauly D (1980). **On the interrelationships between natural mortality, growth parameters, and mean environmental temperature in 175 fish stocks**. J du Conseil Int pour l’Exploration de la Mer.

[39] Hoenig JM (1983). **Empirical use of longevity data to estimate mortality rates**. Fisheries Bull.

[40] Jensen AL (1996). **Beverton and Holt life history invariants result from optimal trade-off of reproduction and survival**. Can J Fisheries Aquat Sci.

[41] Heupel M, Simpfendorfer C (2002). **Estimation of mortality of juvenile blacktip sharks,*****Carcharhinus limbatus*****, within a nursery area using telemetry data**. Can J Fisheries Aquat Sci.

[42] Knip DM, Heupel MR, Simpfendorfer CA (2012). **Mortality rates for two shark species occupying a shared coastal environment**. Fisheries Res.

[43] Newman SP, Handy RD, Gruber SH (2007). **Spatial and temporal variations in mangrove and seagrass faunal communities at Bimini, Bahamas**. Bull Mar Sci.

[44] McKane AJ, Newman TJ (2004). **Stochastic models in population biology and their deterministic analogs**. Phys Rev Lett.

[45] McKane AJ, Newman TJ (2005). **Predator-prey cycles from resonant amplification of demographic stochasticity**. Phys Rev Lett.

[46] McKane AJ, Nagy JD, Newman TJ, Stefanini MO (2007). **Amplified biochemical oscillations in cellular systems**. J Stat Phys.

[47] van Kampen NG (1992). Stochastic processes in physics and chemistry.

[48] Ward-Paige CA, Keith DM, Worm B, Lotze HK (2012). **Recovery potential and conservation options for elasmobranchs**. J Fish Biol.

[49] Peterson I, Wroblewski JS (1984). **Mortality rate of fishes in the pelagic ecosystem**. Can J Fisheries Aquat Sci.

[50] Morrissey JF, Gruber SH (1993). **Habitat selection of juvenile lemon shark*****Negaprion brevirostris***. Copeia.

[51] Guttridge TL, Gruber SH, Franks B, Kessel ST, Gledhill KS, Uphill J, Krause J, Sims DW (2012). **Deep danger: intra-specific predation risk influences habitat use and aggregation formation of juvenile lemon sharks*****Negaprion brevirostris***. Mar Ecol Prog Ser.

[52] Ziemba RE, Myers MT, Collins JP (2000). **Foraging under the risk of cannibalism leads to divergence in body size among tiger salamander larvae**. Oecologia.

[53] Dennis B, Desharnais RA, Cushing JM, Henson SM, Costantino RF (2001). **Estimating chaos and complex dynamics in an insect population**. Ecol Monogr.

[54] Hartig F, Calabrese JM, Reineking B, Wiegand T, Huth A (2011). **Statistical inference for stochastic simulation models – theory and application**. Ecol Lett.

[55] Pimm SL, Redfearn A (1988). **The variability of population densities**. Nature.

[56] Jennings DE, DiBattista JD, Stump KL, Hussey NE, Franks BR, Grubbs RD, Gruber SH (2012). **Assessment of the aquatic biodiversity of a threatened coastal lagoon at Bimini, Bahamas**. J Coastal Conserv.

[57] Heupel MR, Carlson JK, Simpfendorfer CA (2007). **Shark nursery areas: concepts, definition, characterization and assumptions**. Mar Ecol Prog Ser.

[58] Manire CA, Gruber SH: **A preliminary estimate of natural mortality of age-0 lemon sharks,*****Negaprion brevirostris*****.** NOAA Technical Report 115, NMFS 1993

[59] DiBattista JD, Feldheim KA, Garant D, Gruber SH, Hendry AP (2011). **Anthropogenic disturbance and evolutionary parameters: a lemon shark population experiencing habitat loss**. Evol Appl.

[60] Franks BR: **The spatial ecology and resource selection of juvenile lemon sharks (*****Negaprion brevirostris*****) in their primary nursery areas****.** *Ph.d. diss.*, Drexel University, Philadelphia: Pennsylvania; 2007

[61] Newman SP, Handy RD, Gruber SH (2010). **Diet and prey preference of juvenile lemon sharks*****Negaprion brevirostris***. Mar Ecol Prog Ser.

[62] R Development Core Team (2011). R: A Language and Environment for Statistical Computing.

[63] Wiegand T, Jeltsch F, Hanski I, Grimm V (2003). **Using pattern-orientated modeling for revealing hidden information: a key for reconciling ecological theory and application**. OIKOS.

[64] Grimm V, Revilla E, Berger U, Jeltsch F, Mooji WM, Railsback SF, Thulke HH, Weiner J, Wiegand T, DeAngelis DL (2005). **Pattern-orientated modeling of agent-based complex systems: lessons from ecology**. Science.

[65] Anadón JD, Wiegand T, Giménez A (2012). **Individual-based movement models reveals sex-biased effects of landscape fragmentation on animal movement**. Ecosphere.

